# Increased mortality of acute respiratory distress syndrome was associated with high levels of plasma phenylalanine

**DOI:** 10.1186/s12931-020-01364-6

**Published:** 2020-04-30

**Authors:** Jing Xu, Tingting Pan, Xiaoling Qi, Ruoming Tan, Xiaoli Wang, Zhaojun Liu, Zheying Tao, Hongping Qu, Yi Zhang, Hong Chen, Yihui Wang, Jingjing Zhang, Jie Wang, Jialin Liu

**Affiliations:** 1 Department of Critical Care Medicine, Ruijin Hospital, Shanghai Jiao Tong University School of Medicine, Shanghai, China; 2 Department of Pulmonary and Critical Care Medicine, Center of Respiratory Medicine, China-Japan Friendship Hospital, Beijing, China; 3 Department of Pulmonary Medicine, Ruijin Hospital, Shanghai Jiao Tong University School of Medicine, Shanghai, China; 4 Department of Emergency Medicine, Ruijin Hospital, Shanghai Jiao Tong University School of Medicine, Shanghai, China; 5 Department of Gynecology and Obstetrics, Ruijin Hospital, Shanghai Jiao Tong University School of Medicine, Shanghai, China; 6 Department of Biochemistry and Molecular Cell Biology, Shanghai Jiao Tong University School of Medicine, Shanghai, China

**Keywords:** Acute respiratory distress syndrome, Metabolomics, Phenylalanine, Phenylacetylglutamine, Phenylalanine metabolism, Metabolites

## Abstract

**Background:**

There is a dearth of drug therapies available for the treatment of acute respiratory distress syndrome (ARDS). Certain metabolites play a key role in ARDS and could serve as potential targets for developing therapies against this respiratory disorder. The present study was designed to determine such “functional metabolites” in ARDS using metabolomics and in vivo experiments in a mouse model.

**Methods:**

Metabolomic profiles of blood plasma from 42 ARDS patients and 28 healthy controls were captured using Ultra-high performance liquid chromatography tandem mass spectrometry (UHPLC-MS/MS) assay. Univariate and multivariate statistical analysis were performed on metabolomic profiles from blood plasma of ARDS patients and healthy controls to screen for “functional metabolites”, which were determined by variable importance in projection (VIP) scores and *P* value. Pathway analysis of all the metabolites was performed. The mouse model of ARDS was established to investigate the role of “functional metabolites” in the lung injury and mortality caused by the respiratory disorder.

**Results:**

The metabolomic profiles of patients with ARDS were significantly different from healthy controls, difference was also observed between metabolomic profiles of the non-survivors and the survivors among the ARDS patient pool. Levels of Phenylalanine, D-Phenylalanine and Phenylacetylglutamine were significantly increased in non-survivors compared to the survivors of ARDS. Phenylalanine metabolism was the most notably altered pathway between the non-survivors and survivors of ARDS patients. In vivo animal experiments demonstrated that high levels of Phenylalanine might be associated with the severer lung injury and increased mortality of ARDS.

**Conclusion:**

Increased mortality of acute respiratory distress syndrome was associated with high levels of plasma Phenylalanine.

**Trial registration:**

Chinese Clinical Trial Registry, ChiCTR1800015930. Registered 29 April 2018, http://www.chictr.org.cn/edit.aspx?pid=25609&htm=4

## Background

As a common cause of death in patients enrolled in intensive care units (ICUs), acute respiratory distress syndrome (ARDS) is associated with high morbidity (10.4%) and mortality (ranging from 35 to 40%) [1]. Unfortunately, despite many efforts made toward its diagnosis and treatment, the development of a drug treatment for ARDS remains challenging. To pursue the precise care of patients with ARDS, a better understanding of the mechanisms and accurate methods to prognosticate ARDS are required. To date, hundreds of biomarkers have been explored but very few of them have been able to successfully guide the diagnose and treatment of ARDS.

Metabolites are the downstream products of multiple intracellular biomolecules including genes and protein transporters, which enable metabolomics to serve as a remarkable tool that precisely describes “what is happening” within our body [2]. Previous studies have performed metabolomic analysis of plasma, pulmonary edema fluid and bronchoalveolar lavage fluid (BALF) in ARDS patients, preliminarily results revealed a broad range of metabolites that could help in diagnosis and stratify ARDS [3–6]. However, it is still unknown whether there exist any specific metabolites that not only help in identifying different phenotypes of ARDS but also have a crucial function in the disease process. ARDS is characterized by dysregulated immune response and diffused alveolar damage. It is well reported that some metabolites are capable of inducing inflammation and regulating the activation of immune cells [7, 8]. Kentaro Tojo et al. have revealed that the enhancement of glycolysis attenuated the lung tissue injury by protecting alveolar epithelial cells from decline in energy [9]. Their results also indicated that metabolites played an important role in ARDS.

Therefore, our study aimed to find “functional metabolites” that could be potential therapeutic targets in ARDS. To this end, we conducted metabolomics analysis and in vivo experiments in a mouse model of ARDS. Blood plasma samples of patients with ARDS and healthy controls were collected, and metabolomics analysis was conducted to find differential metabolites and altered pathways that are associated with the ARDS mortality. Such functional metabolites were determined by selecting for the ones that were differentially expressed between ARDS patients and healthy controls and between the disease survivors and the non-survivors. Moreover, the mouse model of ARDS was established to investigate the role of identified “differential metabolites” in ARDS mortality.

## Methods

### Study population and sample collection

The study received approval from Ruijin Hospital Ethics Committee of Shanghai Jiao Tong University School of Medicine. All patients who met the criteria for acute respiratory distress syndrome (ARDS) according to the Berlin definition were considered for study enrollment [10]. Patients were excluded if:1) they were less than< 18 years of age; 2) they had any autoimmune diseases; 3) they were on another clinical trial; 4) they had chronic respiratory ailments; Blood samples for metabolomic analysis were collected within 48 h of ARDS diagnose. Volunteers from the Health Center of the Ruijin Hospital of Shanghai Jiao Tong University School of Medicine were selected as healthy controls. Altogether, a total of 42 patients and 28 healthy controls were enrolled in this study. Blood samples that were each 2 mL in volume were collected using heparin tube from ARDS patients and healthy controls. The samples were preserved as previously described [4]. Briefly, all samples were incubated at room temperature for 30 min and then centrifuged at 500 g, 4 °C for 10 min, the supernatant is obtained and stored at − 80 °C till further use. This trial was registered with the Chinese Clinical Trial Registry under the identification number, ChiCTR1800015930.

### Sample preparation

The detail of methods for sample preparation referred to previous study [11]. Briefly, 100 μL of sample was transferred to an Eppendorf (EP) tube and vortexed for 30 s with the addition of 300 μL of methanol (containing 1 μg/mL internal standard). To precipitate proteins, the sample was sonicated for 10 min in ice-water bath and incubated for 1 h at − 20 °C. The sample was then centrifuged at 12000 rpm for 15 min at 4 °C. The resulting supernatant was transferred to a fresh glass vial for analysis. The quality control (QC) sample was prepared by mixing an equal volume of aliquot from the supernatant of each sample. For targeted UHPLC-MS/MS, a 50 μL aliquot of BALF sample was transferred to an Eppendorf tube. Two hundred μL of extraction solution (acetonitrile-methanol, 1:1, and containing isotopically-labelled internal standard mixture) was added and then the samples were vortexed for 30 s, and sonicated for 5 min in ice-water bath. The samples were incubated at − 40 °C for 1 hour and centrifuged at 12000 rpm at 4 °C for 15 min. An 80 μL aliquot of the supernatant was transferred to an auto-sampler vial for UHPLC-MS/MS analysis.

### Untargeted metabolomics detection by UHPLC-MS/MS

Ultra-high Pressure Liquid Chromatography-Mass Spectrum/Mass Spectrum (UHPLC-MS/MS) analysis was performed using an UHPLC system (1290, Agilent Technologies) with a UPLC HSS T3 column (2.1 mm × 100 mm, 1.8 μm) coupled to Q Exactive mass spectrometer (Orbitrap MS, Thermo). The mobile phase A was consist of positive (0.1% formic acid in water)and negative modes (5 mmol/L ammonium acetate in water). The mobile phase B was acetonitrile. The LC method used for detailed metabolite profiling with higher resolution was as follows: column temperature 35 °C, flow rate 0.5 mL/min, injected volume 3 μL. The parameters for elution gradient was as follows: 0 ~ 1.0 min, 1% B; 1.0 ~ 8.0 min, 1% ~ 99% B; 8.0 ~ 10.0 min, 99% B; 10.0 ~ 10.1 min, 99% ~ 1% B; 10.1 ~ 12 min, 1% B. The QE mass spectrometer (Orbitrap MS, Thermo) was used to acquire MS/MS spectra data under the control of the acquisition software (Xcalibur 4.0.27, Thermo). In information-dependent acquisition (IDA) mode, the acquisition software continuously evaluated the full scan MS spectrum. The ESI source conditions were set as following: sheath gas flow rate as 45 Arb, Aux gas flow rate as 15Arb, capillary temperature 400 °C, full MS resolution as 70,000, MS/MS resolution as 17,500, collision energy as 20/40/60 eV in NCE mode, spray Voltage as 4.0 kV (positive) or − 3.6 kV (negative), respectively.

### Targeted amino acids detection by UHPLC-MS/MS

To prepare the Standard Solution Preparation, stock solutions were prepared by dissolving or diluting each standard substance to a final concentration of 10 mmol/L. An aliquot of the stock solutions was transferred to a 10 mL flask to form a mixed working standard solution. A series of calibration standard solutions were then prepared by stepwise dilution of this mixed standard solution (containing isotopically-labelled internal standard mixture in identical concentrations with the samples). The UHPLC system used was the same as described above, equipped with a Waters ACQUITY UPLC BEH Amide column. An Agilent 6460 triple quadrupole mass spectrometer (Agilent Technologies), equipped with an AJS electrospray ionization (AJS-ESI) interface, was applied for assay development. The MRM parameters for each of the targeted analytes were optimized using flow injection analysis. Several most sensitive transitions were used in the MRM scan mode to optimize the collision energy for each Q1/Q3 pair. Among the optimized MRM transitions per analyte, the Q1/Q3 pairs that showed the highest sensitivity and selectivity were selected as ‘quantifier’ for quantitative monitoring. The additional transitions acted as ‘qualifier’ for the purpose of verifying the identity of the target analytes. Agilent Mass Hunter Work Station Software (B.08.00, Agilent Technologies) was employed for MRM data acquisition and processing.

### Data analysis

The raw data were converted to the mzXML format using ProteoWizard and processed by the XCMS-based R-script for peak detection, extraction, alignment, and integration. Then the Kyoto Encyclopedia of Genes and Genomes (KEGG, http://www.genome.jp/kegg/) and Human Metabolome Database (HMDB, http://www.hmdb.ca/) were applied in metabolite annotation.

### Mouse model and survival curve

C57BL/6 mice (8–10 weeks old) were intratracheally injected with *Pseudomonas aeruginosa* [2 × 10^6^ colony-forming units (CFU) of PAO1 strain, ATCC, Manassas, VA, USA] in 50 μL phosphate-buffered saline (PBS) or just equal volume of PBS as a control. To determine the role of Phenylalanine in the mortality of ARDS, mice were pretreated with Phenylalanine (Sangon Biotech, Shanghai, China; A610422–0100) or PBS (10 mg/ml in a total volume of 100ul by intravenous route) 24 h before the intratracheal injection of PAO1, mortality was monitored for 7 days and every 24 h during the week the mice were administrated with another dose of Phenylalanine or PBS until death.

### Assessment of lung injury

The mice were pretreated with Phenylalanine 12 h before the intratracheal injection of PAO1. Every 12 h the mice were given another dose of Phenylalanine and then sacrificed 24 h later after the infection. BALF and lung tissue were obtained to determine the lung injury. The lungs were perfused with 1.5 mL of PBS (3 times, 0.5 mL/perfusion) using a 20-gauge endotracheal catheter, followed by the collection of BALF from the right lung (the left lung was ligated with string). The supernatant of BALF samples was used to assess the protein concentration by bovine serum albumin protein assay (Sigma-Aldrich, St. Louis, MO, USA) and the red blood cell in pellet was removed by lysis buffer (ACK Lysis Buffer, Gibco, Grand Island, NY, USA) and then assayed for white cell counts with a cell counter (Jimbio, Jimbio Technology, Jiangsu, China). The left lung of the mice was processed for hematoxylin and eosin (HE) staining.

### Statistical analysis

Univariate Analysis and multivariate statistical analysis performed by Metabo Analyst (v 4.0) were used to discriminate significant metabolites between different groups. All data were normalized to sum and pareto scaled prior to further analysis. Principle Component Analysis (PCA) was applied to find the distribution features of the dataset. Partial Least Square-Discriminant Analysis (PLS-DA) was used to determine the variable importance in projection (VIP) of each compound, the models were validated by permutation test (*n* = 2000) to avoid over-fitting. Cross validation was to determine the optimal number of components needed to build the PLS-DA mode. Only compounds with a *p* value < 0.05 (student’s T test) and VIP value > 1.0 were considered significantly different between groups. The Pathway analysis module was performed based on KEGG database to identify the utmost affected pathway. Receiver operating characteristic (ROC) curve and area under the ROC curve (AUROC) performed by Graphpad prism (Version 8.0) were used to evaluate the prognostic value of potential biomarkers in patients with ARDS. The combined model of biomarkers was created by binary logistic regression analysis. The independent samples t-test and Mann-Whitney U-test were performed by SPSS 19.0 to compare normally or non-normally distributed data respectively. Categorical data were compared using the chi-square or Fisher’s exact test. Kaplan-Meier plots and the log-rank test were used to compare survival between the groups treated with PBS or Phenylalanine. All tests were two-tailed, *P* < 0.05 was considered to indicate statistical significance.

## Results

### Patient characteristics

Between May 2018 and June 2019, a total of 42 patients who fulfilled the Berlin definition of ARDS and 28 healthy volunteers were included in this study. The patients of ARDS were divided into survivors and non-survivors based on intensive care unit (ICU) mortality. Table 1 shows characteristics of each trial subject at the time of plasma collection including age, gender, risk factors of ARDS, severity scores of disease, laboratory results and outcomes. Appropriate measures were taken to have no difference of age and gender between the ARDS patients and the healthy controls. Compared to the survivors, the non-survivors of ARDS patients had a higher Acute Physiology and Chronic Health Evaluation II (APACHEII) score, other than that, no significant differences were found between the two groups.

**Table 1:** Demographics of ARDS patients and healthy controls

	Controls	Acute respiratory distress syndrome			*P* value
		Total	Survivors	Non-survivors	
Numbers	28	42	27	15	
Age (year)	62.3 ± 15.3	64.2 ± 14.0	63.07 ± 15.5	66.3 ± 10.9	0.48
Gender, Female, n (%)	17 (60.7%)	28 (66.7%)	19 (70.4%)	9 (69.2%)	0.36
**Risk factors of ARDS, n(%)**					
Pneumonia		27 (64.3%)	18 (66.7%)	9 (60%)	0.74
Aspiration		1 (2.4%)	0 (0%)	1 (6.7%)	0.38
Extrapulmonary sepsis		6 (14.3%)	4 (14.8%)	2 (13.3%)	0.64
Pancreatitis		48 (8.9%)	3 (11.1%)	1 (6.7%)	0.55
Trauma or hemorrhagic shock		2 (4.8%)	1 (3.7%)	1 (6.7%)	0.59
others		2 (4.8%)	1 (3.7%)	1 (6.7%)	0.59
**The severity of ARDS**					
Mild		7 (16.7%)	4 (14.8%)	3 (20%)	0.69
Moderate		24 (57.1%)	19 (70.4%)	5 (18.5%)	0.02#
Severe		11 (26.2%)	4 (14.8%)	7 (25.9%)	0.07
APACHE II score		17.1 ± 6.5	15.6 ± 6.1	19.7 ± 6.7	0.05*
PaO2/FiO2		153.5 ± 68.3	167.6 ± 62.9	128.1 ± 72.3	0.07
CRP, mg/dL		97.3 ± 69.5	100.0 ± 63.0	91.9 ± 83.0	0.73
PCT, ng/mL		46.5 ± 84.8	52.9 ± 93.0	34.9 ± 69.4	0.52
Leukocytes, × 10^9^/L		12.6 ± 5.9	13.1 ± 5.8	11.8 ± 6.1	0.50
Neutrophils, ×10^9^/L		11.1 ± 5.4	12.0 ± 5.8	9.4 ± 4.4	0.16
Lymphocytes, ×10^9^/L		2.3 ± 2.9	2.5 ± 3.2	1.9 ± 2.1	0.51
Albumin (g/L)		51.6 ± 53.5	50.8 ± 56.9	52.8 ± 50.4	0.93
Prealbumin (mg/L)		94.6 ± 54.5	100 ± 55.1	86.5 ± 55.0	0.52
Ventilator free days		7.5 ± 13.2	9.6 ± 12.7	3.7 ± 13.7	0.17
ICU stay, days		32.4 ± 26.8	25.8 ± 30.8	23.1 ± 16.9	0.33
Vt/ Bwt		7.03 ± 1.8	7.2 ± 2.0	6.9 ± 1.5	0.72
Inhaled nitric oxide therapy		0	0	0	/
Prone position		4 (9.5%)	4 (14.8%)	0	0.28
ECMO		2 (4.8%)	1 (3.7%)	1 (6.7%)	1.0

Quantitative data are presented as mean ± SD, Qualitative data are presented as number (%), *P*-value for the survivors and non-survivors of ARDS; *APACHE* acute physiologic and chronic health evaluation, *CRP* C-reactive protein, *PCT* procalcitonin, *ECMO* extracorporeal Membrane Oxygenation; * *P* < 0.05 tested by student T test. # *P* < 0.05 tested by chi-square test

### Multivariate data analysis of the metabolites

**Fig. 1:**
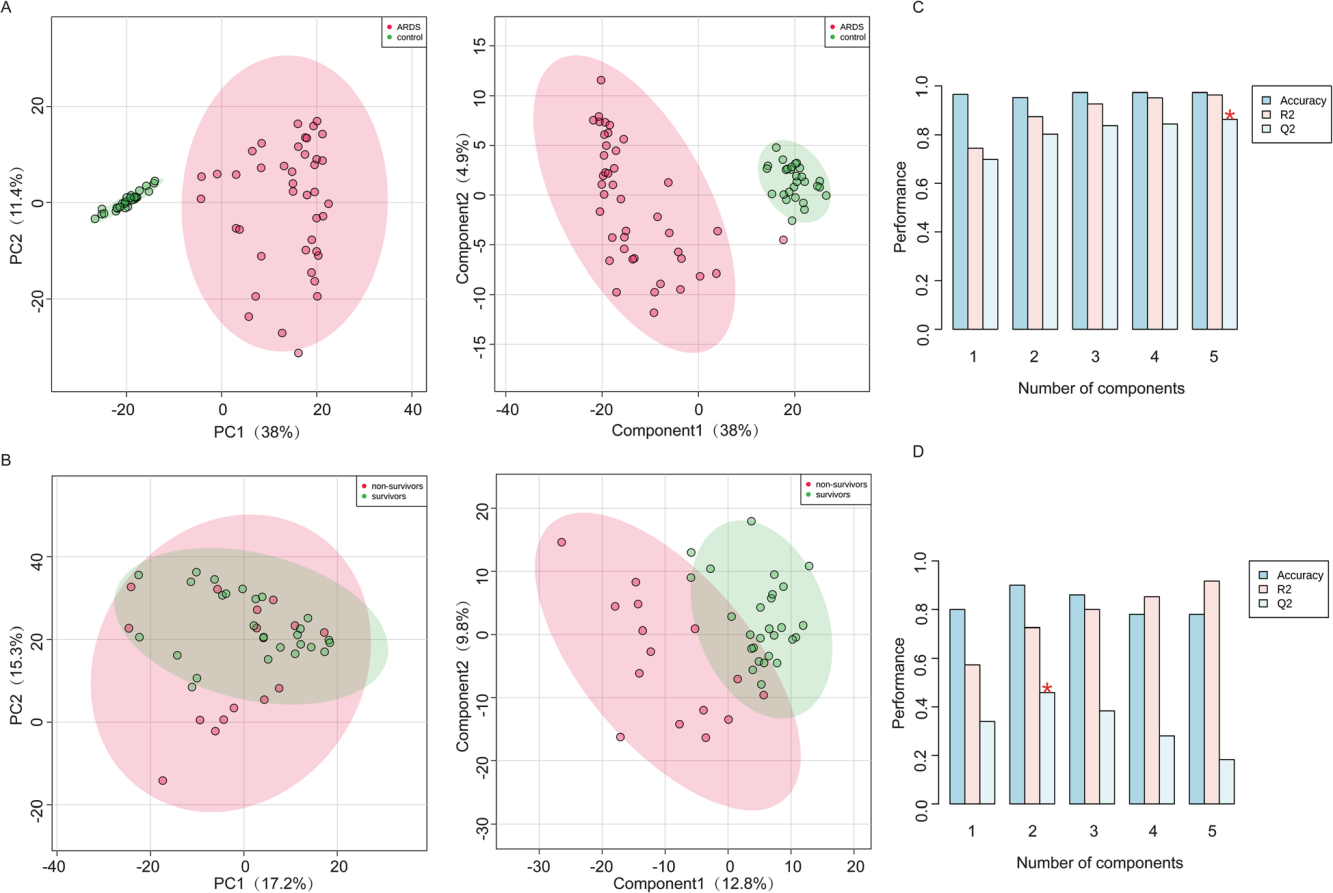
Multivariate data analysis of the metabolites. **a** Principal Component Analysis (PCA) scores plots (left panels) and Partial Least Square-Discriminant Analysis (PLS-DA) scores plots (right panels) of ARDS patients vs healthy controls. Shaded areas are the 95% confidence regions of each group. **b** PCA and PLS-DA scores plots of the survivors vs the non-survivors of ARDS. Shaded areas are the 95% confidence regions of each group. **c** The cross validation test for PLS-DA model of the ARDS patients vs the healthy controls. **d** The cross validation test for PLS-DA model of the survivors vs non-survivors of ARDS. Deep blue represents the cross-validated R^2^ (also known as Q^2^),pink represent the sum of squares captured by the model (R^2^), and light blue represents the prediction accuracy. Red star indicates the best component number in building the model. In PLS-DA of the ARDS patients vs the healthy controls analysis, *R*^*2*^ = 0.93 and Q^2^ = 0.86, the survivors vs the non-survivors of ARDS analysis, *R*^*2*^ = 0.89 and Q^2^ = 0.49, respectively

We identified 414 metabolites from the plasma samples using Ultra-high performance liquid chromatography tandem mass spectrometry (UHPLC-MS/MS) assay. See Supplementary Table S1 for the abundance and distribution of these metabolites in plasma samples from patients and healthy volunteers. The differential metabolomic profiles between the ARDS patients and the healthy controls, and between the survivors and the non-survivors from the patient pool were obtained via Principal Component Analysis (PCA) and Partial Least Square-Discriminant Analysis (PLS-DA). These score plots displayed a significant separation between ARDS patients and healthy controls (Fig. 1a). Cross validation showed that five components were optimal to build the model, based on which we calculated the related statistics (Fig. 1c). The R^2^ was (0.96) for the validity of PLS-DA model against over-fitting and the predictive ability was described by Q^2^ (0.87). The permutation test (*n* = 2000) with a *P* value less than 0.05 indicated toward good predictive ability of PLS-DA models (Figure S1). Statistically significant separation of metabolomic profiles was also observed between the survivors and non-survivors of ARDS (Fig. 1b), and PLS-DA served as a valid model for discriminating the metabolites (*R*^*2*^ = 0.89, Q^2^ = 0.49) (Fig. 1d).

**Fig. 2:**
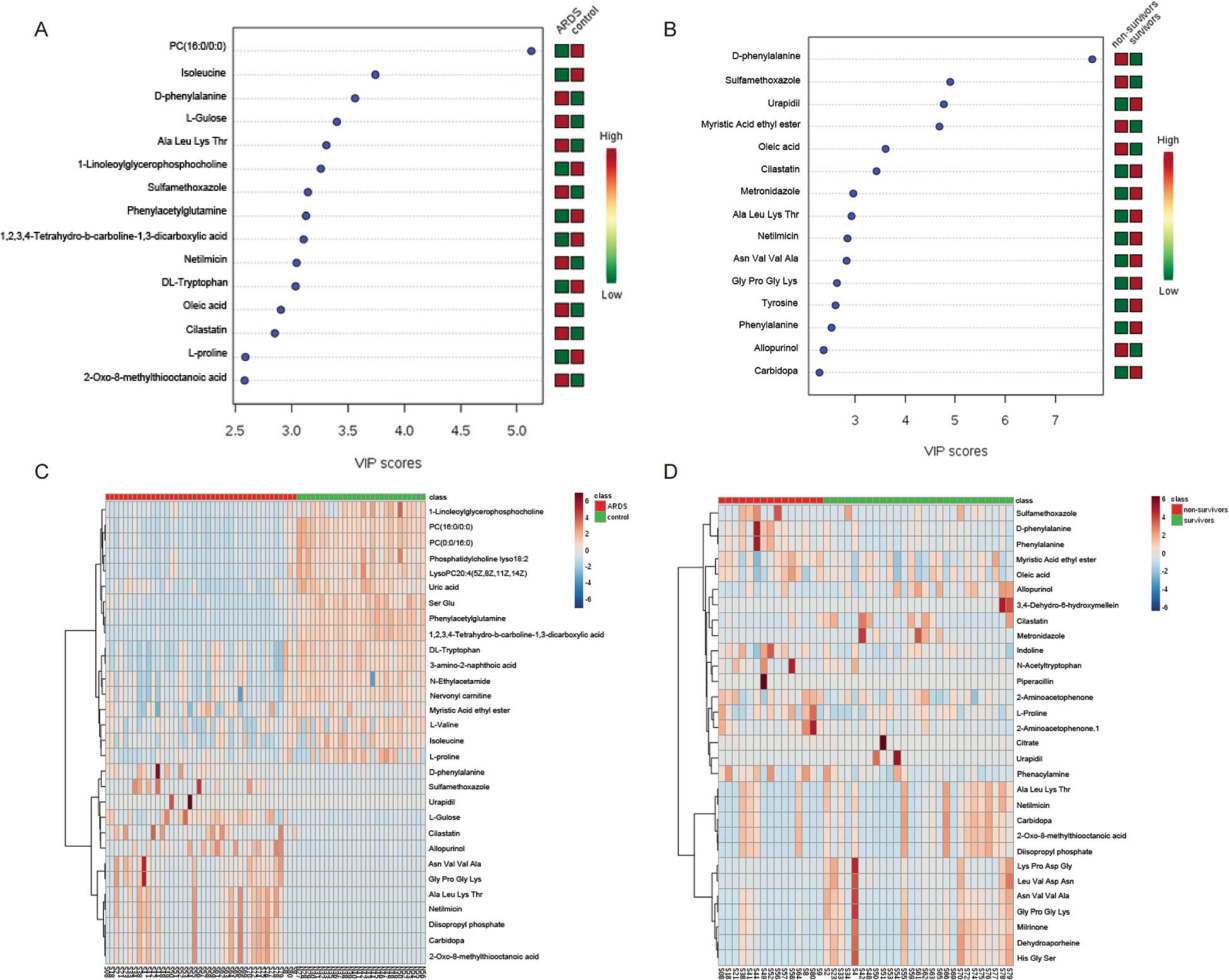
VIP score plot and Heatmap of metabolites. **a** Variable Importance in Projection (VIP) score plot of the metabolites that differed in ARDS patients vs healthy controls. **b** Variable Importance in Projection (VIP) score plot of the metabolites that differed in survivors vs the non-survivors of ARDS. **c** The heatmap showing abundance of the top 30 metabolites based on VIP scores of ARDS patients vs healthy controls. **d** The heatmap showing abundance of the top 30 metabolites based on VIP scores of the survivors vs the non-survivors of ARDS

Variable Importance in the Projection (VIP) is a weighted sum of squares of the PLS loadings taking into account the amount of explained Y-variation, in each dimension (https://www.metaboanalyst.ca). The higher VIP scores of the metabolites had, the more important contribution of it in the differences between groups. A VIP plot generated from the PLS-DA models ranked individual metabolites for their power to discriminate ARDS from controls (Fig. 2a). It can be seen that phosphatidylcholine (PC) (16:0/0:0), isoleucine, D-phenylalnaine and L-Gulose in the plasma were mainly contributed to the metabolic differences between ARDS patients and healthy controls (VIP > 3.0). The differences in the metabolic profiles of the survivors and the non-survivors of ARDS patients are shown in Fig. 2b. They were mainly attributed to D-Phenylalanine, myristic acid and oleic acid in the plasma (VIP > 3.0). The heatmap shows the abundance of top 30 metabolites in all individuals based on VIP scores. We found several glycerophospholipids like 1-Linoleoylglycerophosphocholine, PC(16,0/0:0), Phosphatidylcholine lyso18:2, and LysoPC20:4(5Z,8Z,11Z,14Z) that were significantly downregulated in the ARDS patients compared to the healthy controls (Fig. 2c). The levels of D-Phenylalanine and Phenylalanine increased in the non-survivors compared to the survivors while a group of short peptides containing different amino acid residues downregulated in the non-survivors (Fig. 2d).

### Screening of differentially expressed metabolites as potential mortality predictors for ARDS

**Fig. 3:**
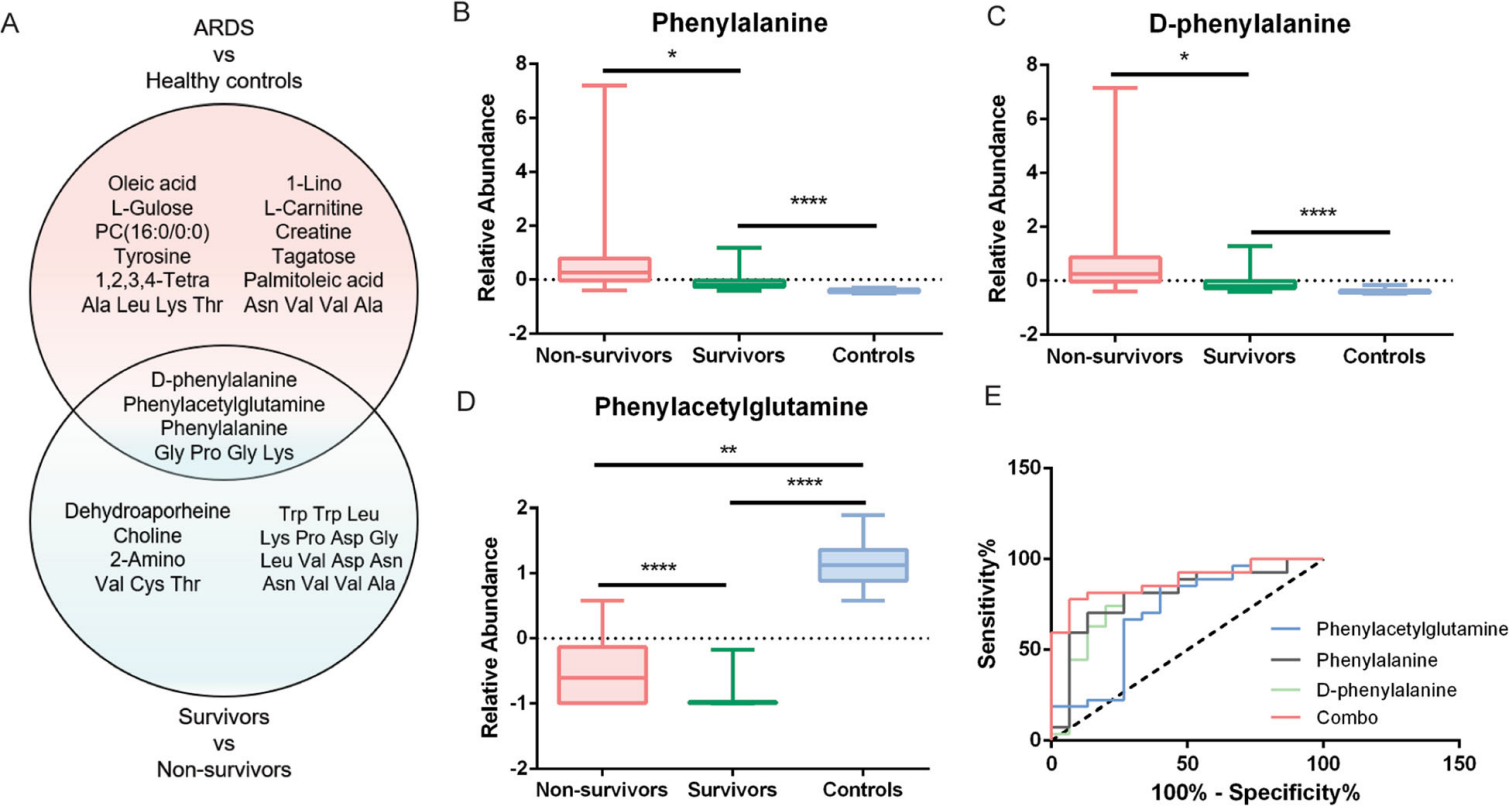
Screening of differentially expressed metabolites as potential mortality predictors for ARDS. **a** The differentially expressed metabolites in both the comparison groups, the pink circle representing the group of ARDS vs healthy controls, and the blue circle representing the group of survivors vs non-survivors. The metabolites in the cross area were those identified in both comparison groups. **b** The box whisker plot of Phenylalanine in the survivors (*n* = 28) and the non-survivors (*n* = 15) of ARDS and healthy controls (*n* = 42). **c** The box whisker plot of D-Phenylalanine in the survivors (*n* = 28) and the non-survivors (*n* = 15) of ARDS and healthy controls (*n* = 42). **d** The box whisker plot of phenylacetylglutamine in the survivors (*n* = 28) and the non-survivors (*n* = 15) of ARDS and healthy controls (*n* = 42). **e** ROC curve of Phenylalanine (AUC = 0.803), D-Phenylalanine (AUC = 0.785) and phenylacetylglutamine (AUC = 0.709) in predicting the mortality of ARDS. In the combined model the area under the curve (AUC) was 0.882. The data was normalized. 1-Lino = 1-Linoleoylglycerophosphocholine; 1,2,3,4-Tera = 1,2,3,4-Tetrahydro-beta-carboline-1,3-dicarboxylic acid; 2-Amino = 2-Aminoacetophenone

We next investigated the potential biomarkers and mortality predictors of ARDS patients. To preliminary screen the differential metabolites, we selected the metabolites that had a *P* value less than 0.05 (calculated by Student’s t-test) and a VIP score greater than 1.0 (calculated using PLS-DA model). The differential metabolites between ARDS patients and healthy controls are displayed in Table 2. Similarly, the metabolites that were found to distinguish the non-survivors from the survivors of ARDS are shown in Table 3. Among all those selected compounds, Phenylalanine, D-Phenylalanine, Phenylacetylglutamine and a short peptide (Gly Pro Gly Lys) were identified in both groups (ARDS vs healthy controls and survivors vs non-survivors) (Fig. 3a). As Phenylalanine, D-Phenylalanine and Phenylacetylglutamine are all involved in the Phenylalanine metabolism, we focused on these three metabolites. The box whisker plots showed that all of three compounds had significant higher concentration in the non-survivors compared to the survivors (Fig. 3b-d). Phenylalanine and D-Phenylalanine had higher levels in the ARDS patients compared to the healthy controls (Fig. 3b-c). We also compared these metabolites in ARDS patients with different severity (mild moderate and severe) according to Berlin’s definition. The levels of Phenylacetylglutamine were markedly lower in moderate ARDS patients compared to severe ARDS patients (Figure S2). There was no significant difference among the concentration of Phenylalanine and D-Phenylalanine in three groups (Figure S2). To investigate the effect of these three differentially expressed metabolites in predicting the mortality of ARDS, we plotted a ROC curve (Fig. 3e) to assess the sensitivity and specificity. The area under the ROC curve (AUC) of Phenylalanine, D-Phenylalanine and Phenylacetylglutamine is 0.803, 0.785 and 0.709, respectively, indicating that Phenylalanine had the highest efficacy in predicting the mortality of ARDS. In the combined model the AUC was 0.882.

**Table 2:** List of the differentiated metabolites between ARDS patients and healthy controls

Name	VI*P* value	*P* value
Oleic acid	4.0374	2.64E-06
D-Phenylalanine	3.4261	2.88E-06
L-Gulose	2.4759	3.78E-10
PC(16:0/0:0)	2.162	1.11E-12
Tyrosine	1.8285	1.73E-05
1,2,3,4-Tetrahydro-beta-carboline-1,3-dicarboxylic acid	1.6314	9.87E-40
Palmitoleic acid	1.6274	3.41E-05
Phenylacetylglutamine	1.5472	1.25E-33
1-Linoleoylglycerophosphocholine	1.2548	4.13E-06
L-Carnitine	1.1955	1.06E-04
Creatine	1.1823	9.14E-04
Tagatose	1.0799	4.05E-05
Phenylalanine	1.0284	2.20E-06
Ala Leu Lys Thr	2.0248	2.41E-05
Asn Val Val Ala	1.2398	0.000491

The exotic drugs were not shown in this table

**Table 3:** List of the differentiated metabolites between survivors and non-survivors of ARDS patients

Name	VIP value	*P* value
D-Phenylalanine	6.0449	0.01706
Phenylacetylglutamine	3.3932	0.001569
Phenylalanine	1.8439	0.011172
Dehydroaporheine	1.8075	0.005813
Choline	1.1339	0.020561
2-Aminoacetophenone	1.1019	0.016276
Val Cys Thr	1.1417	0.0085869
Trp Trp Leu	1.2142	0.034223
Lys Pro Asp Gly	1.8047	0.038664
Leu Val Asp Asn	1.5179	0.025572
Gly Pro Gly Lys	2.2304	0.026714
Asn Val Val Ala	2.4713	0.017745

The exotic drugs were not shown in this table

### Pathway analysis reveals the phenylalanine pathway to be one of the most significantly altered pathways in ARDS patients

**Fig. 4:**
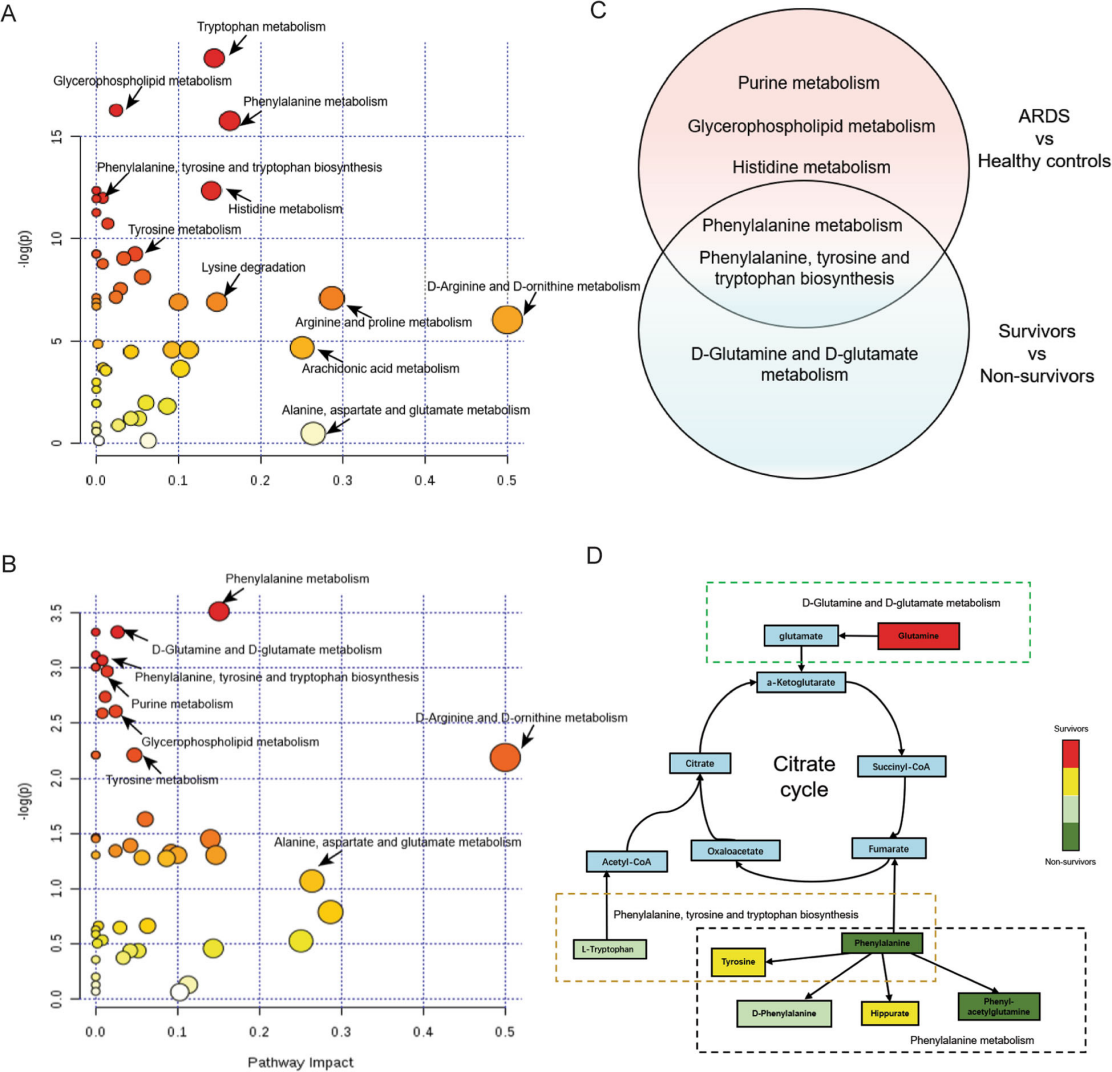
Pathway analysis reveals the Phenylalanine pathway to be one of the most altered pathways in ARDS patients. **a** Pathway analysis uncovered the altered pathways in ARDS patients vs healthy controls. **b** Pathway analysis uncovered the altered pathways in the survivors vs non-survivors of ARDS. All matched pathways were shown according to *P* values from pathway enrichment analysis (y-axis) and all pathway impact values were according to pathway topology analysis (x-axis). The color and size of each circle are based on P values and pathway impact values, respectively. The deeper the red of the nod, the more significant alteration of the pathway is observed. Small P value and big pathway impact circles indicate that the pathway is greatly influenced. **c** The pathways that are altered between ARDS and healthy controls (pink circle) or between survivors and non-survivors (blue circle). The pathways in the cross area were identified in both groups. **d** Schematic diagram of metabolic pathway networks. The metabolites involved in the selected pathways (*P* < 0.05,impact factor > 0) altered between the survivors and the non-survivors were marked in different colors. Light blue means those metabolites are not in my data and are used as background for enrichment analysis; red (*P* < 0.05) and yellow (*P* > 0.05) means the metabolites are upregulated in the survivors deep green (*P* < 0.05) and light green (*P* > 0.05) mean that the metabolites are downregulated in survivors with different levels of significance. The metabolites in the box belong to the same pathway

To gain the functional interpretation of these numerous compounds in ARDS, we applied Pathway Analysis of the metabolites identified in our data. The pathways that altered between ARDS and healthy controls or between survivors and non-survivors were listed in Table S2. Figure 4a and b display all matched pathways. We screened the pathways with an impact factor greater than 0 and a *P* value less than 0.05, the top 5 pathways in the group of ARDS versus healthy controls were 1) purine metabolism pathway, 2) Phenylalanine, tyrosine and tryptophan biosynthesis pathway, 3) histidine metabolism pathway, 4) Phenylalanine metabolism pathway and 5) glycerophospholipid metabolism pathway. In the survivors versus non-survivors group, only three pathways satisfied the standards of screening, they were 1) D-Glutamine and D-glutamate metabolism pathway, 2) Phenylalanine, tyrosine and tryptophan biosynthesis pathway, and 3) Phenylalanine metabolism pathway. Figure 4c shows that in both the ARDS versus healthy controls group and the non-survivors versus survivors group, Phenylalanine metabolism pathway, Phenylalanine, tyrosine and tryptophan biosynthesis pathway were identified as the most significantly altered pathways. To visualize the change of metabolites in the most significantly altered pathways between non-survivors and survivors, the metabolic network was plotted (Fig. 4d), all the matched metabolites in our data that involved in the pathways were marked in red, yellow and green according to the different levels of significance. In Phenylalanine metabolism pathway, five compounds were matched and three of them (Phenylalanine, D-Phenylalanine and phenylacetylglutamine) upregulated while tyrosine and hippurate were downregulated in the non-survivors compared to the survivors. In Phenylalanine, tyrosine and tryptophan biosynthesis pathway, three metabolites were matched with our data, L-Tryptophan and Phenylalanine levels increased while Tyrosine levels decreased in the non-survivors. In D-glutamine and D-glutamate metabolism pathway, only one metabolites (glutamine) was matched and was found to be significantly downregulated in the non-survivors.

### Phenylalanine administration increased the lung injury and mortality of ARDS

**Fig. 5:**
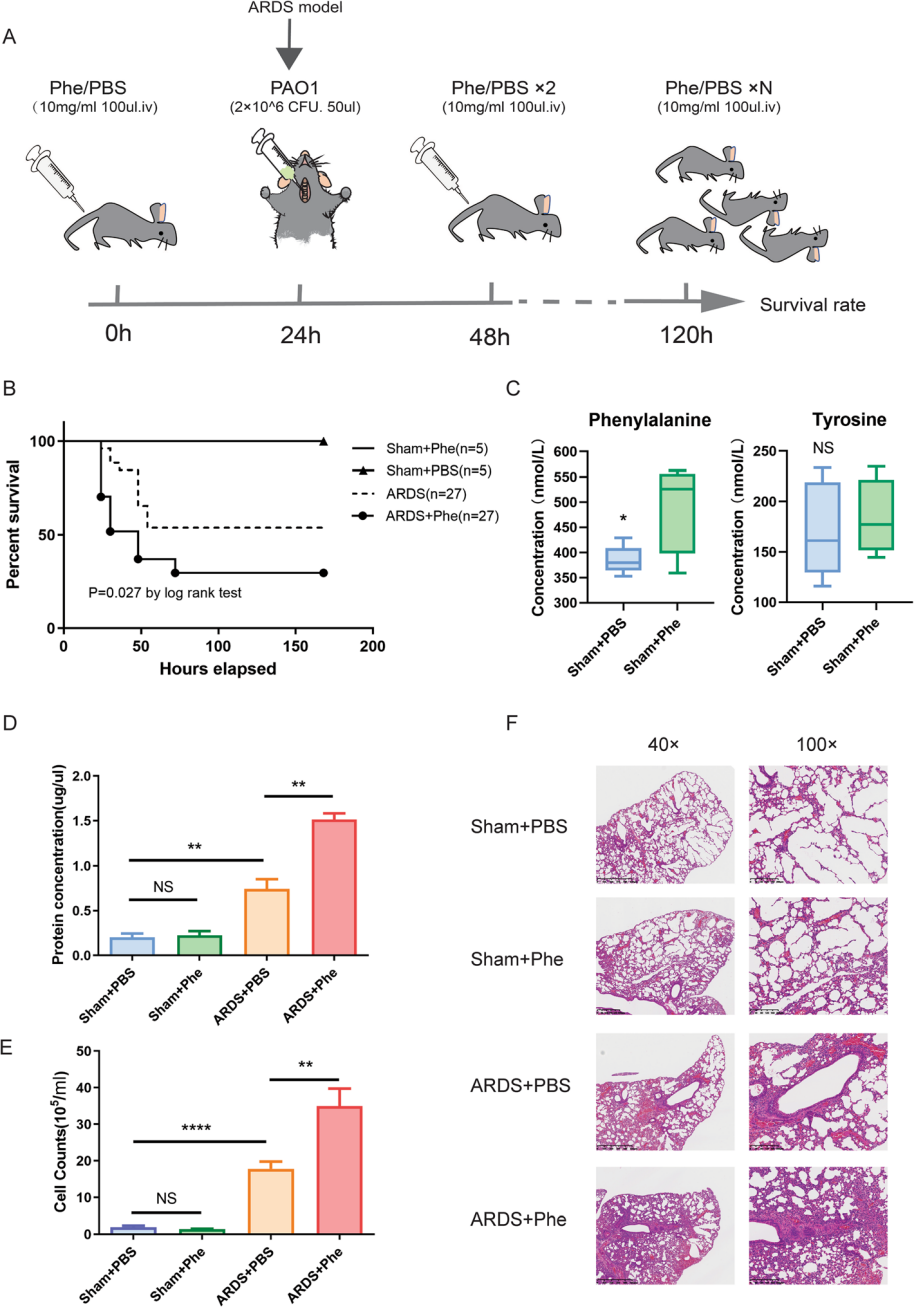
Phenylalanine administration increased the lung injury and mortality of ARDS. **a** The protocol of Phenylalanine administration and ARDS model establishment **b** The survival rate of ARDS mice (*n* = 27/group) and Sham mice (*n* = 5/group) treated with Phenylalanine or PBS. **c** The levels of Phenylalanine and tyrosine in BALF of sham mice treated with Phenylalanine or PBS (*n* = 4–5/group). **d** The protein concentration in bronchoalveolar lavage fluid (BALF) (*n* = 3–5 /group). **e** The white cell counts in BALF (*n* = 3–5 /group). **f** The hematoxylin and eosin staining of lung tissue (*n* = 3–5 /group) Phe = Phenylalanine. Each value represents the mean ± SEM of one of the three independent experiments. Kaplan Meier Survival analysis and comparisons were performed by log-rank test

The differential metabolites and pathway analysis indicated that Phenylalanine, D-Phenylalanine and Phenylacetylglutamine might play a role in ARDS. D-Phenylalanine and Phenylacetylglutamine are products of Phenylalanine, and the ROC analysis showed that Phenylalanine had the highest accuracy of predicting the mortality. Therefore, we investigated whether Phenylalanine increased the mortality of ARDS in a mouse model. The ARDS mouse model was established and Phenylalanine was administrated by intravenous injection. As shown in Fig. 5a, mice were pretreated with Phenylalanine at 0 h and received intratracheal injection of *Pseudomonas aeruginosa* (PAO1) at 24 h. The ARDS mice and sham mice were administrated with either a dose of Phenylalanine or PBS every 24 h, following which the mice started to die at 24 h after injection of PAO1. A significant increase of mortality rate was observed around 54 h, at which time 48% of the mice injected with Phenylalanine and 30% of mice injected with PBS found dead. At 7 days after PAO1 injection, the Phenylalanine group had significantly lower survival rate (8%) compared to those treated with PBS (14%) (Fig. 5b). The same amount of Phenylalanine was administrated in sham mice without ARDS every 24 h for 7 days and no death was observed during the period (Fig. 5b), confirming that the concentration of Phenylalanine we used was not lethal in control mice. To determine the changes of Phenylalanine and other related amid acids in BALF after the Phenylalanine injection, we performed targeted UHPLC-MS/MS to assay Phenylalanine, glutamine, tyrosine and tryptophan in sham mice after two doses of intravenous injection of Phenylalanine. The Phenylalanine in BALF significantly increased after injection, whereas the tyrosine (downstream products of Phenylalanine) did not change, indicating that intravenously injection of Phenylalanine increased the levels of this amino acid in lung. We found the levels of glutamine and tryptophan (not significantly) in BALF also increased after injection of Phenylalanine (Figure S3). The lung injury of mice were assayed 24 h post injection with PAO1, protein and white cell counts in bronchoalveolar lavage fluid (Fig. 5b-c) increased in ARDS mice injected with Phenylalanine compared to those treated with PBS, indicating that Phenylalanine increased the recruitment of inflammatory cells and impairment of alveolar epithelial cells. The pathological changes in lungs of mice with ARDS assayed by hematoxylin-eosin (HE) staining indicated severer destruction of alveoli and inflammation in Phenylalanine group than that in PBS group (Fig. 5e). The lung injury in sham mice treated with Phenylalanine and PBS had no significant differences. Together, these results confirmed that Phenylalanine administration increased the lung injury and mortality of ARDS mice but not in sham mice.

## Discussion

In this study, we found that the global metabolomic profile of blood plasma from an ARDS patient was remarkably different than that of a healthy volunteer. Striking difference in the metabolomic profile was also noticed between the survivors and the non-survivors of this disease. By metabolomics analysis, we uncovered metabolites differences as well as the altered pathways that attributed to the mortality of ARDS patients. Of note, we showed that the level of Phenylalanine increased in the non-survivors compared to the survivors and we confirmed using a mouse model that Phenylalanine administration increased the lung injury and mortality of mice with ARDS.

Phenylalanine is an essential amino acid required for biosynthesis of neurotransmitters [12]. The high serum Phenylalanine is neurotoxic, producing intellectual disability, and other neurologic features [13]. Recently, the role of Phenylalanine in acute inflammatory diseases has been increasingly investigated. It was found that the serum Phenylalanine increased in patients post trauma or with sepsis [14] and was associated with the activation of immune response [15, 16]. In a recent study, Shie-Shian Huang and et al. uncovered that high levels of serum Phenylalanine was associated with high mortality risk in patients with severe infection [17]. Consistent with our findings of Phenylalanine, Akhila Viswan and his colleagues reported that the relative concentration of Phenylalanine increased in non-survivors of ARDS patients compared to survivors [4]. Another study also revealed the upregulation of Phenylalanine metabolism in patients with more severe ARDS compared to those with less severity. Although these studies provided supportive evidence of the associations between Phenylalanine and the prognosis of ARDS, it is hard to determine whether the increased Phenylalanine was just a phenotype reflecting the severity or it also played a role in the process of ARDS [3]. Through the injection of Phenylalanine in ARDS mice, our study demonstrated that Phenylalanine played a detrimental role of in ARDS.

The accumulation of Phenylalanine could be a consequence of reduced activity of Phenylalanine 4-hydroxylase (PHA) and its cofactor 5,6,7,8-tetrahydrobiopterin (BH4) [18]. These enzymes convert Phenylalanine into tyrosine. To be noted that, in our results, the levels of tyrosine decreased in the non-survivors compared to the survivors of ARDS patients (not a significant difference though), which means the turnover of Phenylalanine into tyrosine in the non-survivors may be impaired. A number of studies have indicated that the deficiency of PHA and BH4 in inflammatory disease may be due to the overwhelming production of reactive oxygen species (ROS) [12, 19]. Therefore, it was possible but not conclusive that the increased Phenylalanine in the non-survivors of ARDS was due to the deficiency of PHA and BH4 induced by inflammation.

The accumulated Phenylalanine in turn amplified the already existing inflammation. A study found that intraperitoneal injection of Phenylalanine increased IL-2 secretion in early pregnancy mice, which implied that Phenylalanine could sustain T cell proliferation and thereby enhance the adaptive immune in response [20]. More recently, Ming Jiang et.al demonstrated that Phenylalanine enhanced the innate immune response of the host [21]. Multiple immunological processes involving neutrophils, macrophages, and dendritic cells participate in mediating lung tissue injury in ARDS [22]. Thus the activation of immune response by Phenylalanine may exert negative impact on ARDS. Our data showed that Phenylalanine increased the lung injury of mice with ARDS, which we speculated was associated with the role of Phenylalanine in promoting inflammation.

Phenylacetylglutamine was recently reported to be a gut microbiota generated metabolite fermented from dietary Phenylalanine [23]. The levels of phenylacetylglutamine significantly decreased in human after a 7-day course of oral broad spectrum antibiotics cocktail. Further investigation revealed microbial porA gene in Clostridium facilitated the conversion of Phenylalanine into phenylacetylglutamine [23]. In our study, the levels of phenylacetylglutamine were lower in ARDS than that in controls, which was presumably due to the damage of gut microbiota caused by antibiotics use in ARDS patients. Phenylacetylglutamine was demonstrated to enhance platelet activation-related phenotypes and thrombosis potential in animal models of arterial injury [23]. Pulmonary thrombosis is common in sepsis-induced ARDS shown by human and animal studies [24]. Platelets could promote pulmonary vascular damage in sepsis induced ARDS, thereby aggravating the lung injury [25]. We found the non-survivors of ARDS had higher levels of phenylacetylglutamine than survivors, the mechanisms underlying may be related to the roles of phenylacetylglutamine in modulating platelets and thrombosis.

In the last decades, a broad range of drug therapies emerged for improving ARDS, but none showed efficacy in phase II and III trials [26–31]. Given the high mortality rate of ARDS, even a small improvement can save many lives. Nutritional input may be a good choice as a new adjuvant strategy for ARDS, since most metabolites can be manipulated by simply controlling the uptake from food. Omega-3 fatty acids were once used for treatment of ARDS but ended up with a negative results, one of the reasons for this could be the failure of identifying specific metabolites in ARDS patients [32]. However, with the development of metabolomics, we are able to screen the most specific metabolites that play a role in ARDS [2]. Our study has revealed that controlling the uptake of Phenylalanine might be a novel strategy for treating ARDS. This would also open new avenues in the control of pathological inflammatory responses. Sufficient (high-dose) protein was suggested to be provided in critically ill patients [33], nevertheless, eight essential amino acids including Phenylalanine were indispensable in our commonly used parenteral and enteral nutrition. To reduce the uptake of Phenylalanine, the Phenylalanine-restricted diet designed for phenylketonuria patients might be a good choice. It is a feasible and easy approach that can be implemented in ARDS patients.

The present study still has some limitations. The small samples size of our ARDS patients was the main one. However, the matched age and gender ratio as well as the strict screening standards by multivariate and univariate analysis minimized the artificial mistakes. Secondly, the commonly used drugs in critically ill individuals could also be detected in our analysis. It was difficult to interpret the role of these metabolites, therefore, we didn’t include them in our list of differential metabolites and focused only on the endogenous compounds. Thirdly, the nutritional status of ARDS and control subjects was not controlled for, which could affected the results of metabolomics. Lastly, the untargeted screening by metabolomics can only obtain relative concentrations of metabolites, quantitative metabolomics should be used in the future studies to confirm the absolute concentration of Phenylalanine and its related metabolites in ARDS patients.

## Conclusions

In conclusion, our study revealed that the perturbance of Phenylalanine metabolism was associated with the rate of mortality in ARDS. Phenylalanine increased in the ARDS patients compared to the healthy controls, and in the non-survivors compared to the survivors of ARDS. Moreover, our study is the first to demonstrate high levels of Phenylalanine were associated with the aggravated lung injury and increased mortality of ARDS mice.

## Supplementary information

**Additional file 1.**

**Additional file 2.**

**Additional file 3.**

## Data Availability

The datasets used and/or analyzed during the current study are available from the corresponding author on reasonable request.

## Abbreviations

ARDS: Acute respiratory distress syndrome
ICU: Intensive care unit
BALF: Bronchoalveolar lavage fluid
QC: Quality control
UHPLC-MS/MS: Ultra High Pressure Liquid Chromatography -Mass Spectrum/ Mass Spectrum
KEGG: Kyoto Encycloa of Genes and Genomes
HMDB: Human Metabolome Database
APACHE: Acute physiologic and chronic health evaluation
CRP: C-reactive protein
PCT: Procalcitonin
PCA: Principal component analysis
PLS-DA: Partial least square-discriminant Analysis
VIP: Variable importance in projection
HE: Hematoxylin-eosin staining
PHA: Phenylalanine 4-hydroxylase
BH4: 5,6,7,8-tetrahydrobiopterin
