# Supplementary material for: Increased mortality of acute respiratory distress syndrome was associated with high levels of plasma phenylalanine
Source: Respir Res. 2020 Apr 30;21:99. doi: 10.1186/s12931-020-01364-6 (PMC7193408; doi:10.1186/s12931-020-01364-6)
Supplement: Supplementary file 3 — Additional file 3. [file 12931_2020_1364_MOESM3_ESM.docx]

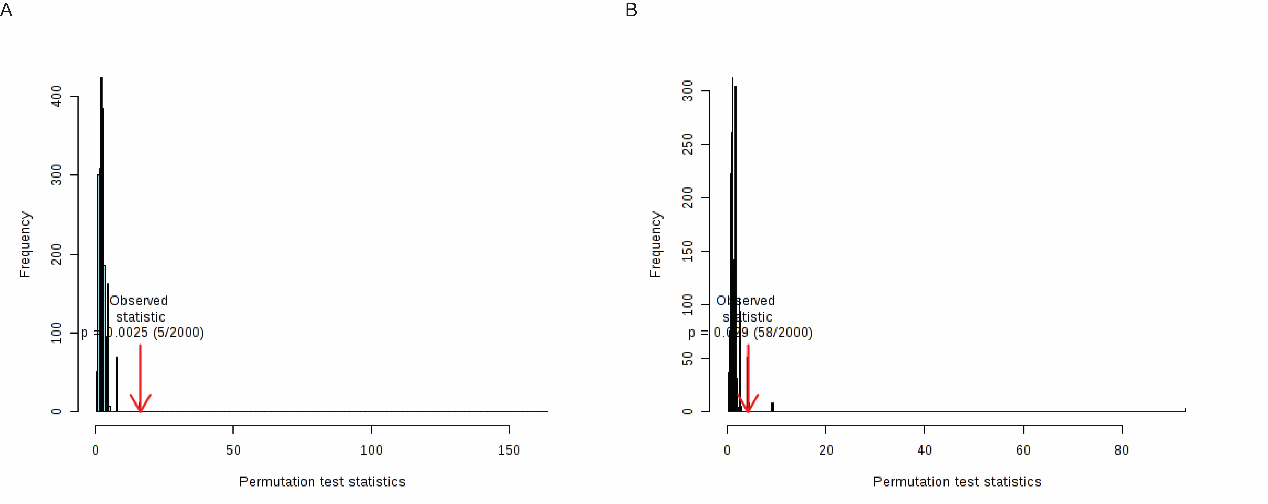
**Supplementary:**

FigureS1 (A) The permutation test for PLS-DA model of the ARDS patients vs the healthy controls (B) The permutation test for PLS-DA model of survivors vs the non-survivors of ARDS.


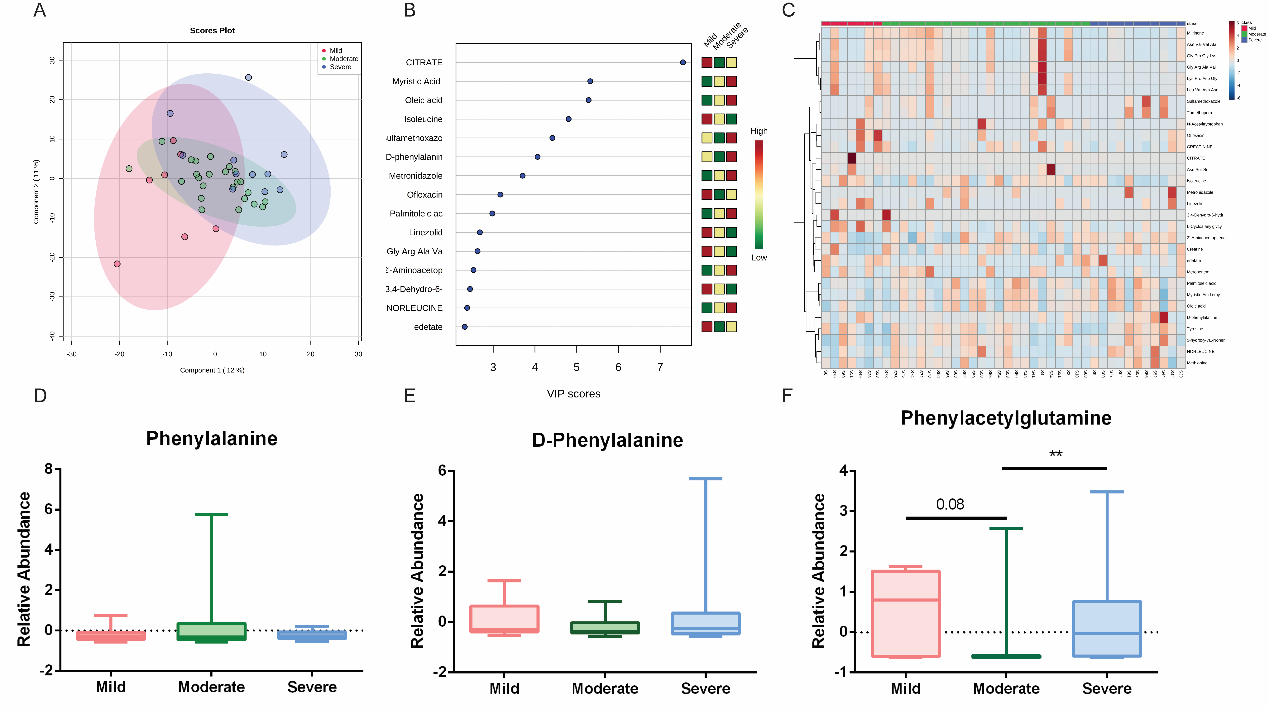


**FigureS2.**(A) The PLS-DA plot of mild, moderate and severe ARDS patients. (B) The
